# Indocyanine green based antimicrobial photodynamic therapy as an adjunct to non-surgical periodontal treatment in periodontal maintenance patients: a clinico-microbiological study

**DOI:** 10.12688/f1000research.133230.2

**Published:** 2024-08-20

**Authors:** Urbashi Roy Chowdhury, Deepa Kamath, Pooja Rao, Suchitra Shenoy M, Ramya Shenoy

**Affiliations:** 1 Ex-Post graduate Trainee, Periodontology, Manipal College of Dental Sciences, Mangalore, Manipal Academy of Higher Education, Manipal, Karnataka, 575001, India; 2 Professor, Periodontology, Manipal College of Dental Sciences, Mangalore, Manipal Academy of Higher Education, Manipal, Karnataka, 575001, India; 3 Assosiate Professior, Microbiology, Kasturba Medical College, Mangalore, Manipal Academy of Higher Education, Manipal, Karnataka, 575001, India; 4 Professor and Head, Microbiology, Kasturba Medical College, Mangalore, Manipal Academy of Higher Education, Manipal, Karnataka, 575001, India; 5 Professor and Head, Public Health Dentistry, Manipal College of Dental Sciences, Mangalore, Manipal Academy of Higher Education, Manipal, Karnataka, 575001, India

**Keywords:** Periodontal disease, Chronic periodontitis, Residual periodontal pockets, Photodynamic therapy, Laser Therapy, Indocyanine Green, Maintenance therapy, Photosensitizers.

## Abstract

*Background:* Antimicrobial Photodynamic therapy for the treatment of periodontitis is being increasingly gaining attention but at present, very limited data are available on the clinical and microbiological outcomes obtained following Indocyanine Green as the photosensitizer in Maintenance patients. The objective was to evaluate the efficiency of Indocyanine(ICG)-green based photodynamic therapy as an adjunct to scaling and root planing in patients enrolled in maintenance therapy.

*Methodology:* Using a split mouth study design, 24 participants enrolled in the maintenance therapy, having diagnosed as Periodontitis, were randomly subjected to scaling and root planing(SRP). The test group additionally received ICG-based (Aurogreen ^®^, Aurolabs, Madurai, India,1mg/ml) aPDT with an 810nm diode laser. Clinical assessment of Plaque index, modified Sulcus bleeding index, Probing pocket depth, Clinical loss of attachment and microbiological analysis of *A. actinomycetemcomitans*, *P. gingivalis, T. forsythia* and *F.nucleatum* were performed at baseline and 3 months after treatment.

*Results:* It was observed that although there was no significant difference between the test and control group at baseline and 3 months, there was a statistically significant reduction in the mean values in both the groups at 3 months. Microbiological analysis showed substantial reduction in detection frequency of the bacteria assessed at 3 months in both the groups.

*Conclusion:* Within the limits of the study, ICG-based aPDT did not show additional advantage over SRP alone at 3 months, though it could be a promising treatment modality in maintenance patients in terms of patient comfort and the treatment time taken. More randomised clinical trials should be employed to understand the exact mode of action of ICG based aPDT and its role in treatment of periodontal disease.

## Introduction

Periodontitis is a result of an exaggerated reaction of the host tissue in response to the microbial challenge which ultimately leads to loss of hard and soft tissues of the periodontium. ^1^ With this sort of a continuous microbial challenge, the periodontal tissues are subjected to toxic bacterial (virulence) factors that have the potential to alter various host cell operations. Periodontal therapy is primarily aimed at removal of these hard tissue and soft tissue deposits from the root surface to prevent progression of the disease. This is achieved by means of scaling and root planing (SRP) by hand and/or power driven instruments. ^2^ However, in spite of being the standard of care for treatment of periodontal disease, SRP may not be sufficient to completely remove all periodontal pathogens. ^3^ The varied clinical presentations such as deep pockets, presence of furcations or intrabony defects may render mechanical debridement inadequate for disruption of the tenacious biofilm which is the primary niche of putative periodontal pathogens.

LASER technology have been around for quite some time now and is currently in the 4 ^th^ decade in the dental field. It holds a promising non-invasive mode of treatment when it comes to periodontal therapy. ^4^ As discussed previously, periodontal disease causing bacteria not only accumulates on the hard tissue but also can invade the soft tissues of the periodontium. In this regard, Diode lasers have proven to be a boon because they are nothing but soft tissue lasers which have an affinity for pigment containing cells for example black pigmented bacteria (spirochetes). ^5^ LASER also exerts an antibacterial effect which may be further improved by the addition of a photosensitizing dye. This phenomenon is known as photodynamic therapy (PDT). ^6^ Indocyanine green is one such photosensitizer which absorbs wavelength at a range of 750-950 nm with a maximum value of 810 nm and exerts its main action by photo-thermal effect which induces cell damage by increasing intracellular temperature. ^7^ PDT includes a photochemical reaction mediated by oxygen which gets activated on application of light in the presence of a photosensitizing compound that leads to generation of highly reactive nascent oxygen species that are toxic to the microorganisms. The Major advantages of antimicrobial PDT (aPDT) are that they explicitly target cells, causing no collateral damage to non-pigmented host tissue cells and is only initiated when laser light is irradiated. It also lacks the development of resistant bacterial species, which is common with injudicious use of antibiotics. ^6^

Residual pockets may contain periodontal pathogens persistently and may even re-harbour these microorganisms which are incompatible with periodontal health. ^8^ Therefore, such sites need special professional attention. Re-instrumentation repeatedly in these sites may cumulatively cause damage to the hard tissues, thereby calling for maintenance protocols for residual pockets which are efficient even after repeated application. ^9^

Recent body of evidence have shown that adjunctive use of PDT with SRP for treatment of residual periodontal pocket may provide additional improvement and better outcomes. However, there is no available literature which have evaluated the efficacy of the treatment protocol using indocyanine green as a photosensitizer in photodynamic therapy in maintenance patients. Therefore, the purpose of this study is to assess the changes in clinical and microbiological parameters following multiple applications of PDT using indocyanine green and diode laser along with SRP in treating patients enrolled in periodontal maintenance programme.

## Methods

### Patient population and study design

The present split-mouth, double-blinded, randomized controlled clinical trial comprised of 25 patients suiting the inclusion criteria. They were recruited from Department of Periodontology, Manipal College of Dental Sciences, Mangalore, India from December 2021 to January 2023.

### Ethical approval

The study protocol was reviewed and approved by Institutional Research Ethics Committee and was conducted in accordance with Helsinki Declaration 1975, as revised in 2013(registered at Clinical Trial Registry India as CTRI/2023/01/61447).

### Informed consent

Informed consent was taken from all participants intimating them the nature, potential risks, and benefits of their participation in the study. They were free to withdraw from the study at their will. The information sheet and consent form can be found as Extended data.

### Sample size calculation

Reference of the present study was taken from Grzech lesniak et al. ^10^ An effect size of 0.2 was considered with a 95% confidence interval and 80% power. Thus, a sample size of 25 was calculated for this split mouth design.

### Inclusion criteria

a) Aged above 18 years
b) Chronic periodontitis with residual pockets of ≥4 mm in two different quadrants of the mouth, after completion of active periodontal treatment and currently under maintenance
c) FMPS ≤ 25% (Full-mouth plaque score)
d) FMBS ≤ 25% (Full-mouth bleeding score)

### Exclusion criteria

a) Systemic illness that would compromise periodontal status
b) patient on immune-depressant, anti-epileptic, calcium antagonist, blood thinners.
c) Smokers (5 cigarette or more per day)
d) Use of systemic antibiotics in the last twelve months
e) Pregnancy

### Allocation of the study groups and Blinding

The subjects were arbitrarily allocated to either of the interventions: A) (SRP) with antimicrobial photodynamic therapy (aPDT) on right side; SRP on left side or B) SRP right side and aPDT on left side. Selected sites were randomly assigned to one of the two treatment modalities by a computer generated block randomization (allocation ratio of 1:1) before each intervention procedure. A third staff member (RS) who wasn’t directly involved in the study carried out the randomization. Opaque sealed envelopes were used to conceal the allocation sequence. The primary investigator, outcome assessor, biostatistician as well as the patients were unaware of the details of the series of the study. As and when a patient came and fit the criteria for the study, the envelope was opened and treatment protocol was followed as described below.

**Treatment protocol**

*Periodontal measurements*

**Figure 1.**
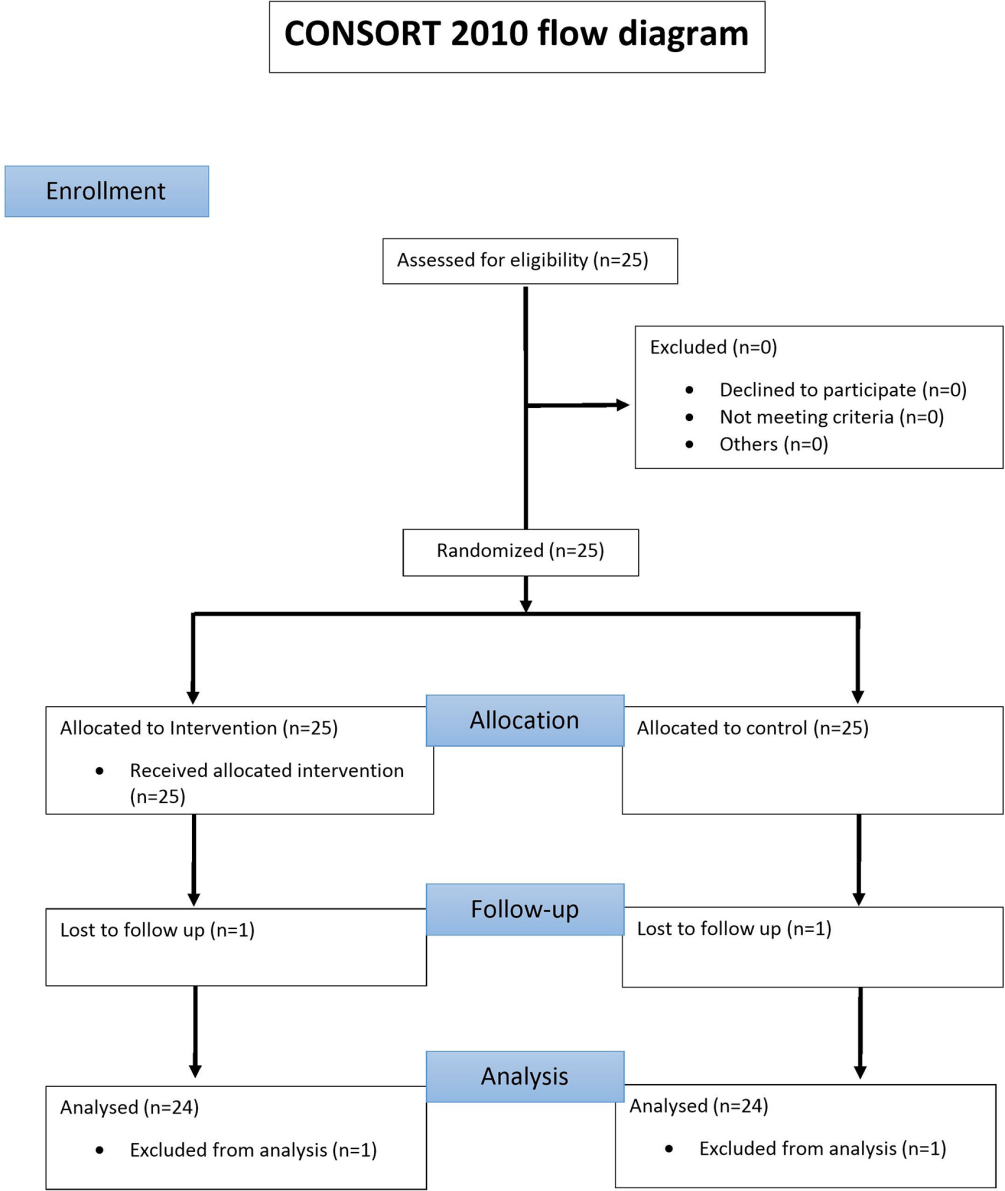
Consort flow diagram reporting the enrolled, treated and evaluated patients.

All 25 Patients underwent full mouth clinical periodontal assessment around all teeth by a single calibrated examiner (DK) following the CONSORT guidelines ( Figure 1: consort flow chart). ^11^ The measurements were taken at four sites per tooth (distal, mesial, facial/buccal, lingual/palatal) using a University of North Carolina No.15 periodontal probe (Hu-friedy™, Chicago IL) and Customized acrylic stents were prepared to guide the probe placement in the same plane. Clinical variables included the measurements of plaque index (PI), modified sulcular bleeding index (mSBI), probing pocket depth (PD) and clinical attachment level (CAL). PD was measured as the distance between the gingival margin and the base of the pocket and CAL was calculated as the distance from the CEJ and the bottom of the sulcus or of the periodontal pocket. The primary outcome being probing depth and clinical attachment level based on the number of sites and secondary outcome being plaque index and bleeding index.

*Preparation of indocyanine green*

Under aseptic circumstances a fresh 5 mg/mL solution with the photosensitizer dye Indocyanine green (Aurogreen ^®^, Aurolabs, Madurai, India) was prepared. In 5ml of sterile water, ICG is dissolved to prepare an initial 5 mg/mL ICG stock solution. This solution is further diluted in saline solution at the ratio of 1:5 to achieve the final ICG concentration of 5 mg/mL. ^12^ The aqueous solution becomes unstable on coming in contact with the environment and hence should be utilised on the same day within 10 hours.

*Laser parameters*

The laser system used in the study is Diode laser (Picasso AMD) with wavelength of 810 nm. It was applied in a continuous mode, circumferentially around the tooth, with a power of 2 W for 60 s. Total energy produced is 6 J/cm ^2^.

*Microbiological assessment*

**Figure 2.**
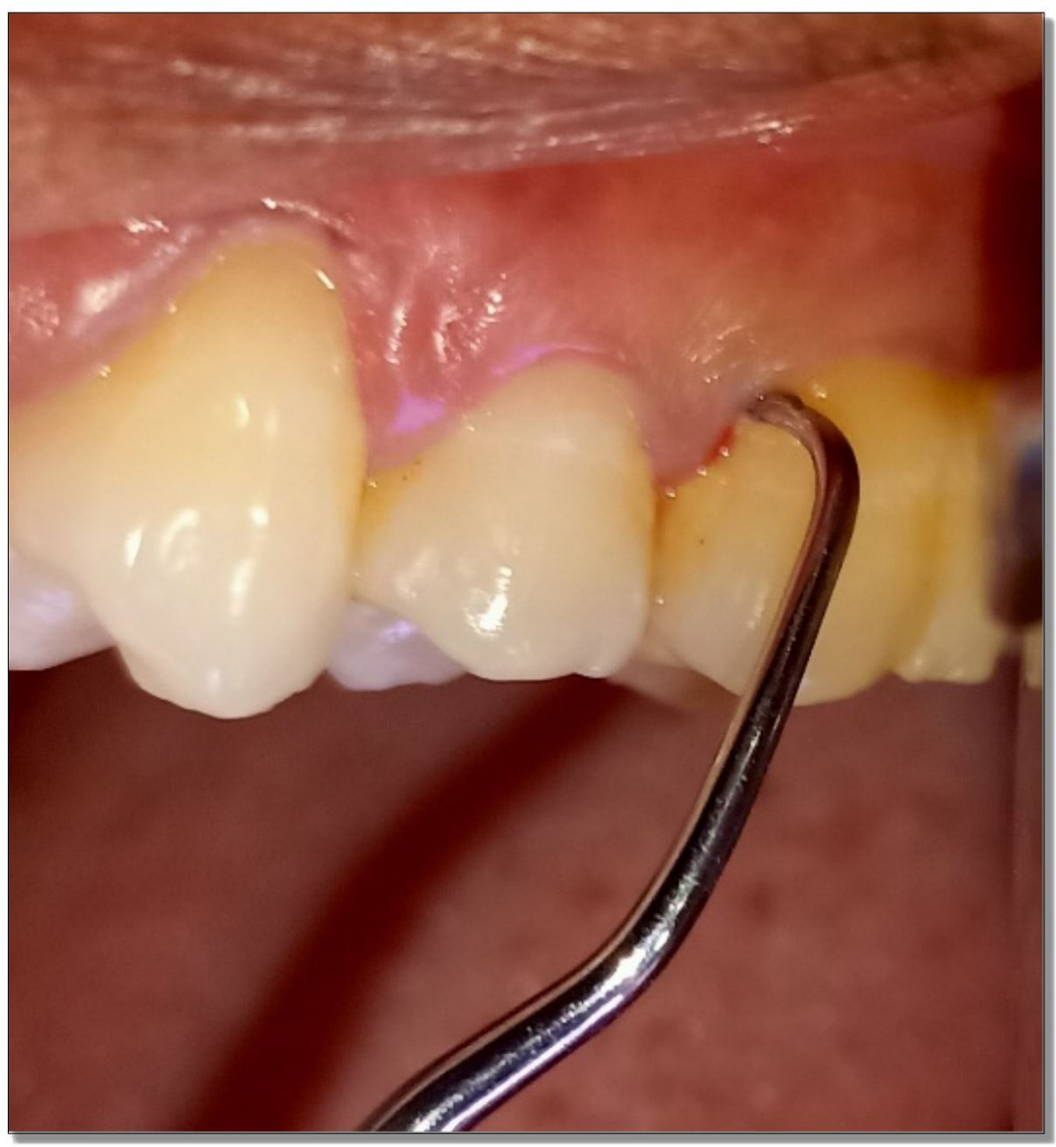
Sample collection with curette.

Microbiological samples were collected through plaque from the deepest periodontal pockets at baseline and three months after therapy using sterile curettes ( Figure 2). They were then transferred to Eppendorf tubes containing 500 μL of sterile phosphate buffer saline (PBS). Microbiological analysis consisted of a molecular test for the detection of four periodontal pathogens. The test was performed with Multiplex PCR (polymerase chain reaction) method. Bacterial DNA was extracted using DNA thermal cycler (ProFlex™ 3× 32-well PCR System, Applied Biosystems, Foster City, CA) following the instructions of the manufacturer ( Table 1).

**Table 1.** Primers used in PCR analysis.

Bacteria	Primer sequence	Basepairs	Thermal conditions
***Aa***	5′-TACTAATTAAGTGGGAAA-3′ 5′-ATCTCTCAGTGTTAATAG-3′	637 basepair	95°C for 15 minutes for Initial denaturation 40 cycles - 95°C for 30 seconds, 60°C for 1 minute, 72°C for 1 minute. 72°C for 10 minutes for final extension
***Pg***	5′-GGAGAACCTACTGGAAAG-3′ 5′-GGAGTTTATCTGGACTTGA-3′	637 basepair	95°C for 15 minutes for Initial denaturation 40 cycles - 95°C for 30 seconds, 60°C for 1 minute, 72°C for 1 minute. 72°C for 10 minutes for final extension
***Tf***	5′-GTTAAGGTAACATTAGGT-3′ 5′-TTTATCGTAGATCAGAAT-3′	600 basepair	95°C for 15 minutes for Initial denaturation 40 cycles - 95°C for 30 seconds, 60°C for 1 minute, 72°C for 1 minute. 72°C for 10 minutes for final extension
***Fn***	5′-CAACACCTAGTAATCATC-3′ 5′-CGAATGCTAATACCTATA-3′	653 basepair	95°C for 15 minutes for Initial denaturation 40 cycles - 95°C for 30 seconds, 60°C for 1 minute, 72°C for 1 minute. 72°C for 10 minutes for final extension

Aa - Aggregatibacter actinomycetemcomitans, Pg - Porphyromonas gingivalis, Tf - Fusobacterium nucleatum, Fn - Tannerella forsythia

The following periodontal pathogenic species were analysed:

• Aggregatibacter actinomycetemcomitans
• Porphyromonas gingivalis
• Fusobacterium nucleatum
• Tannerella forsythia

### Treatment

**Figure 3.**
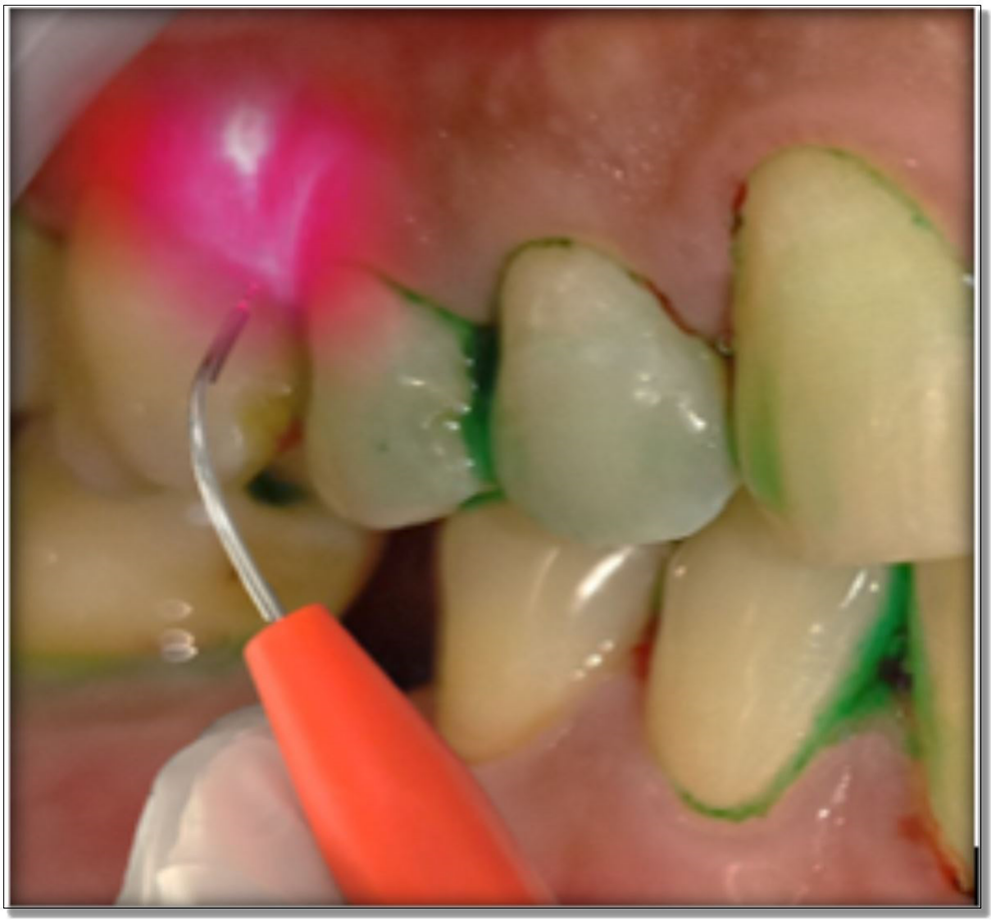
Application of LASER in the test group.

At day zero, patients fulfilling the inclusion criteria were subjected to full mouth SRP. According to the randomisation the test sites were then treated with PDT which is explained as follows. Immediately after full mouth SRP, the test sites were properly dried using air syringe to receive the photosensitizer. 0.5 ml of the dye was then applied in the test site using a syringe. To avoid ‘carry-across’ effect, a conscious effort was made to not spill the photosensitizer in other parts of the oral cavity. Anyhow, the dye is not known to get activated without laser light. Distilled water was used after 2 minutes to wash out the free photosensitizer. The 810 nm diode soft tissue laser was brought in contact with the gingival tissues below the gingival margin and light was applied for 60 seconds in a circumferential manner around the tooth. The laser fibre tip was kept in contact with the soft tissue wall constantly ( Figure 3). The strokes addressed small sections and were systematically overlapping. The fibre tip was continually inspected to remove any accumulated debris with water moistened gauze. aPDT was repeated after 14 days.

The contralateral site received sham LASER which imitated the irradiation without it being turned on. All the patients were monitored for 3 months. They were blinded throughout the procedure and study period to maintain the integrity of the assignment.

### Statistical analysis

Statistical analysis was done for all the clinical parameters and microbiological parameters. Mean and standard deviation was calculated at baseline and at 3 months’ follow-up. Data was analysed using paired ‘t’ test for plaque index, gingival index, clinical attachment level and probing depth at baseline and at 3 months’ follow-up. Mc Nemar P test and Fischer exact P test were used for microbiological parameters. Statistical Package for Social Sciences (SPSS), version 17 (SPSS Inc, Chicago IL) was the used software. A P value of <0.05 was considered significant.

## Results

### General data

This split mouth, single centre, randomised controlled trial was done for a period of 3 months. There were no unwanted events reported by any patient throughout the study. Out of 25 subjects initially recruited, one was lost to drop out and subsequently 24 patients were carried forward for analysis. 10 participants were females and 14 were males, aged between 30 and 78 years. The age and sex distribution of both groups were uniform (P>0.05) ( Table 2). A total of 130 teeth were examined, 66 in test group and 64 in control group, with 171 intervention sites: 86 in test group and 85 in control group.

**Table 2.** Demographic features of study population.

Variable	All N=25	
Age (mean ± SD)	48.1 ± 11.5.	
**Gender**	**N**	**%**
Male	14	58%
Female	10	42%

SD - Standard Deviation.

### Clinical parameters

The participants in the study showed decreased full mouth plaque score (FMPS) and full mouth bleeding score (FMBS) that is <25%. This signifies that all of them had maintained an optimum standard of oral hygiene throughout the study period. The results also showed a substantial reduction of PI, mSBI throughout the study period in both groups with P value of <0.000 ( Table 3).

**Table 3.** Comparison of changes in clinical parameters (in mm) of both groups at different time intervals.

Clinical parameters	Groups	Baseline	3 months	Baseline vs 3 months, P value
PI	Test group	1.7±0.25	1.14±0.23	0.000 *
	Control group	1.65±0.32	1.16±0.18	0.000 *
	P value	0.5 ^†^	0.6 ^†^	
SBI	Test group	1.82±0.24	1.05±0.26	0.000 *
	Control group	1.69±0.42	1.01± 0.15	0.000 *
	P value	0.2 ^†^	0.6 ^†^	
PPD	Test group	5.57±0.21	3.33±0.31	0.000 *
	Control group	5.57±0.24	2.84±0.44	0.000 *
	P value	0.2 ^†^	0.6 ^†^	
CAL	Test group	5.57±0.21	3.37±0.33	0.000 *
	Control group	5.57±0.24	3.14±0.55	0.000 *
	P value	0.8 ^†^	0.6 ^†^	

* Statistically significant at P<0.05.

^†^ Not statistically significant at P>0.05.

There was statistical significance from baseline to 3 months regarding PD with values of 5.57±0.21 mm and 3.33±0.31 mm respectively for the test group and 5.57±0.24 mm and 2.84±0.44 mm respectively for the control group (P value <0.05). Similarly, for gain in CAL in the test group, there was a statistically significant difference from baseline (5.57±0.21 mm) to 3 months (3.37±0.33 mm) and in the control group from baseline (5.57±0.24 mm) to 3 months (3.14±0.55 mm) (P value <0.05). However, intergroup comparison of PD and CAL did not show any statistical significance at any given period (P>0.05) ( Table 3).

### Microbiological parameters

**Figure 4.**
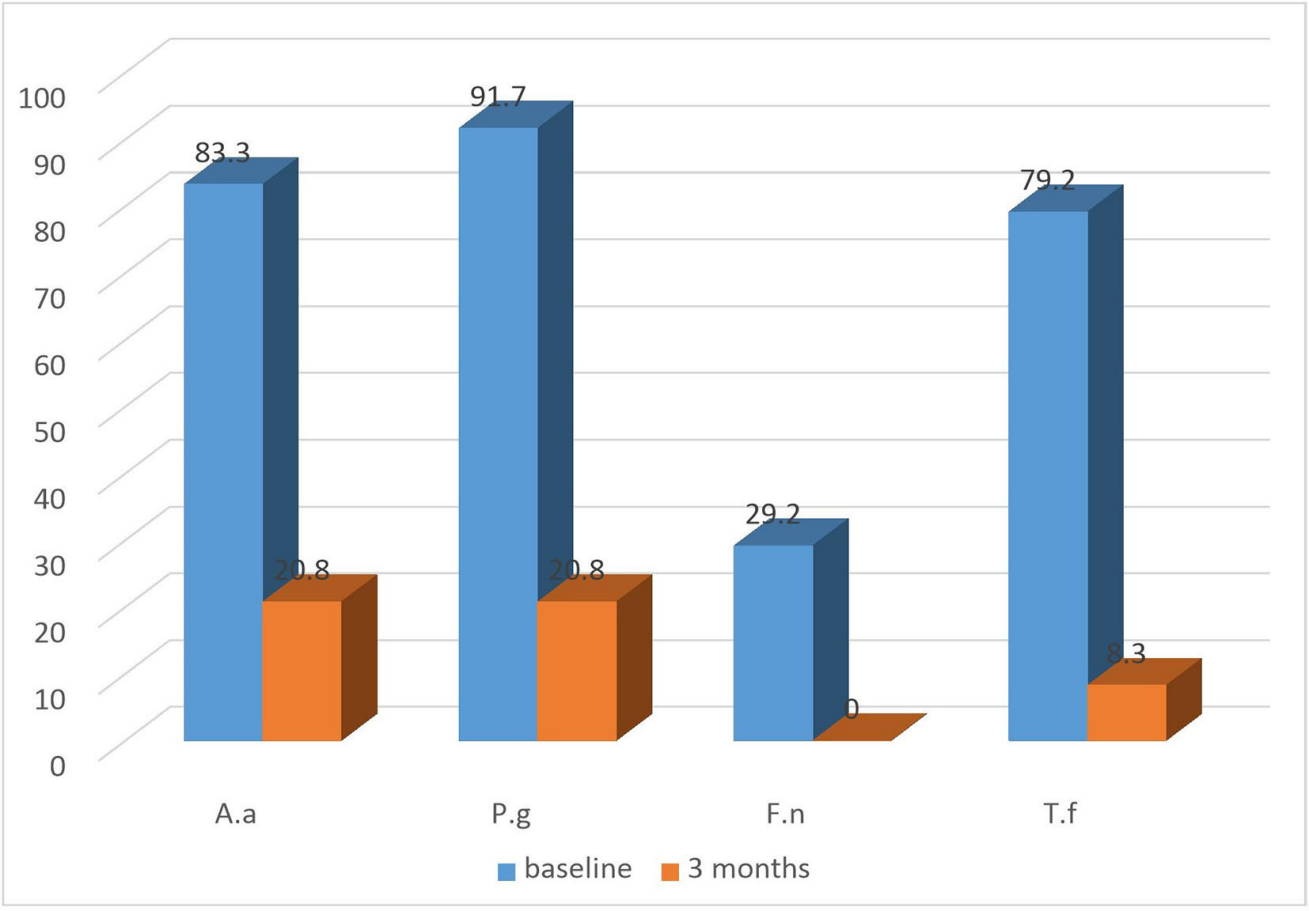
Detection frequency (%) of A.a, P.g, F.n, T.f in Test group at baseline and at 3 months.

**Figure 5.**
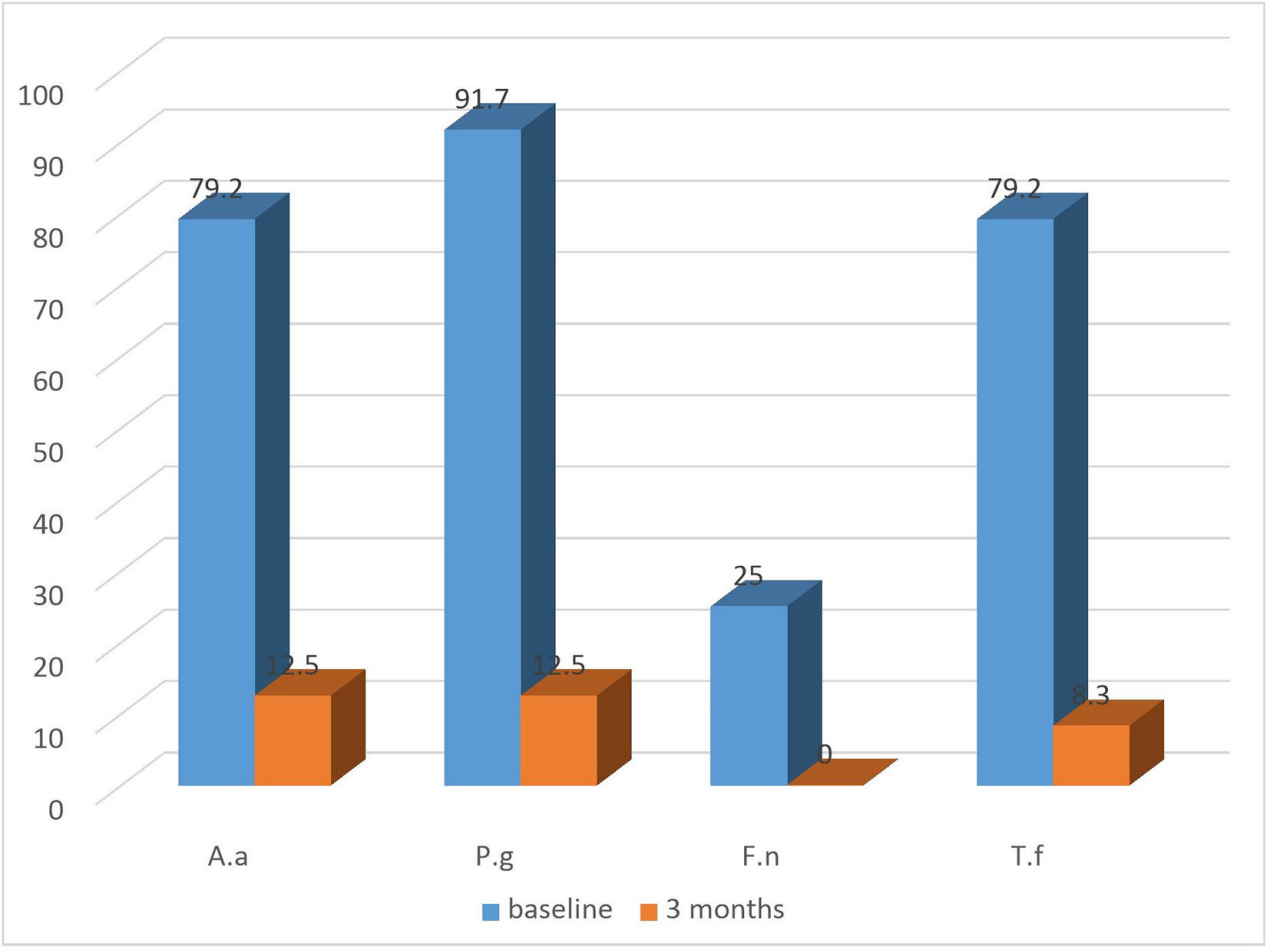
Detection frequency (%) of A.a, P.g, F.n, T.f in control group at baseline and at 3 months.

All groups exhibited a substantial decrease in the detection frequency of *A.a, P.g, T.f, F.n* from baseline till the termination of the study ( Figure 4: test) and ( Figure 5: control). Both the groups showed equally greater reduction in detection frequency of *P.g* and *T.f* at 3 months ( Table 4).

**Table 4.** Detection frequency of organisms at baseline and 3 months.

Pathogen	Time interval	Test group	Control group
		Frequency of organisms present (%)	Frequency (%)
*Aggregatibacter* *Actinomycetemcomitans* *	baseline	20 (83.3)	19 (79.2)
	3 months	5 (20.8)	3 (12.5)
*Porphyromonas gingivalis* *	baseline	22 (91.7)	22 (91.7)
	3 months	5 (20.8)	3 (12.5)
*Fusobacter nucleatum* **	baseline	7 (29.2)	6 (25)
	3 months	0	0
*Tannerella forsythia* *	baseline	19 (79.2)	19 (79.2)
	3 months	2	2 (8.3)

* Using Mc Nemar P test, Detection of frequency of organisms between test and control group was found to be not statistically significant.(p>0.05).

** p value could not be calculated as expected frequency was ‘0’ at 3 months for both the groups.

## Discussion

The corner-stone of cause-related therapy is considered to be non-surgical periodontal therapy (NSPT) which encompasses the removal of supragingival and subgingival biofilm and calculus. ^13^ Yet, a number of ‘residual pockets’ often remain after non-surgical periodontal therapy. ^14^ Residual pockets, particularly >4 mm, have shown to be a positive predictor of clinical loss of attachment over the years and hence represents a clinical scenario which is more difficult to treat than a patient of untreated periodontitis ^15^^.^^16^ Photodynamic therapy has been used in periodontal treatment for over a quarter of a century now. They are being increasingly used as adjunctive treatment with NSPT and eliminates disadvantage such as bacterial resistance seen with systemic or local antibiotics. ^17^

**Figure 6.**
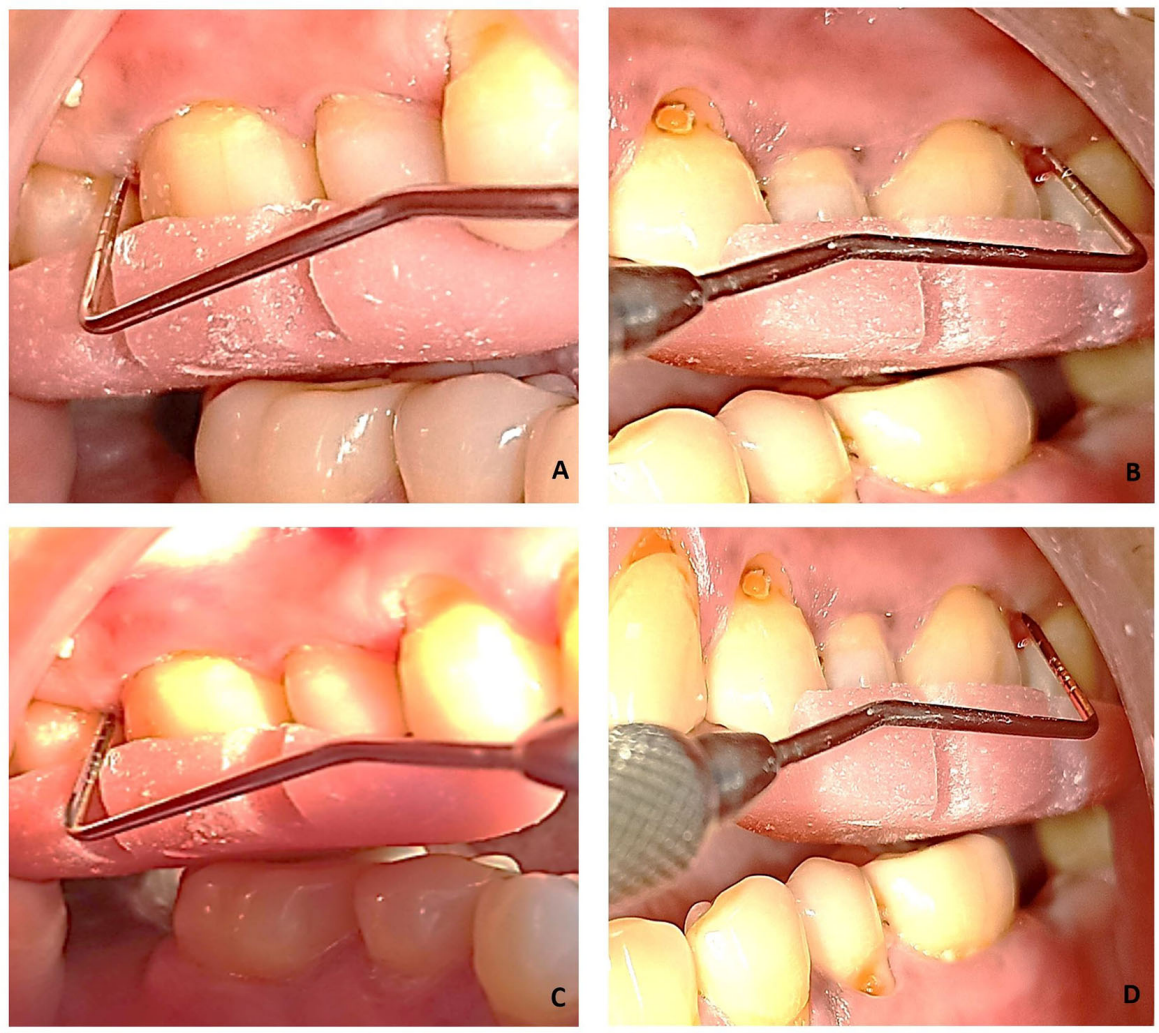
A,Pre-operative probing depth of test site - 5 mm; B, Pre-operative Probing pocket depth of control site – 5 mm; C,3 months post-operative probing pocket depth of test site – 3 mm; D, 3 months post-operative probing pocket depth of control site - 3 mm.

This study focussed on evaluating the clinical and microbiological effects of antimicrobial photodynamic therapy using indocyanine green in patients enrolled in maintenance therapy, having residual pockets. The use of a split mouth design in the present study provided an advantage over parallel design study where the same subject served as control, hence comparison of all treatment methods could be done under similar and optimally standard healing periods. A statistically significant improvement was seen at 3 months for plaque scores and bleeding scores in both the intervention groups without any intergroup differences at any given point. There was a significant reduction in the probing pocket depth and considerable gain in clinical attachment level at the end of 3 months in both groups ( Figure 6). However, the difference between the two groups were not statistically significant. There are studies which are in agreement with the present study ^6^^,^^18^^.^^19^ A systematic review by Azarpazhooh et al. (2010) demonstrated that PDT as a treatment modality whether stand-alone or as adjunct, was not superior to SRP therapy alone. ^20^

However, a systematic review of four RCTs by Xue et al(2017) stated that additional clinical improvement were observed in SRP+PDT group compared to SRP alone for residual pockets for supportive periodontal therapy. ^21^ As none of the studies used Indocyanine green as the photosensitizer, no clear-cut conclusion can be drawn. With that, coming to the next aspect- the photosensitizer, majority of available literature on PDT demonstrate the use of conventional photosensitizers that are methylene blue or toluidine blue. The present randomised controlled trial is the first to determine the outcome of PDT using Indocyanine Green as the photosensitizing agent in treatment of residual pockets in maintenance therapy patients. However, the outcomes in the current study, though favourable at 3 months, did not prove added benefits of ICG compared to SRP alone. This is not commensurate with previous studies utilising ICG as photosensitizing agent. A systematic review conducted by Bashir et al (2021) concluded that ICG mediated PDT in treatment of periodontitis significantly improved the clinical outcomes compared to SRP alone. ^22^ This disparity may be due to the fact that there is no standardised protocol for use of soft tissue diode LASER and the frequency of antimicrobial PDT application for treatment of periodontal disease. Lack of standardisation eliminates many studies for analysis in systematic reviews. Also, the utilisation of ICG in the field of periodontitis is relatively new hence it should be acknowledged that there may have been studies which are yet in the process of trial, or have gone through publication.

**Figure 7.**
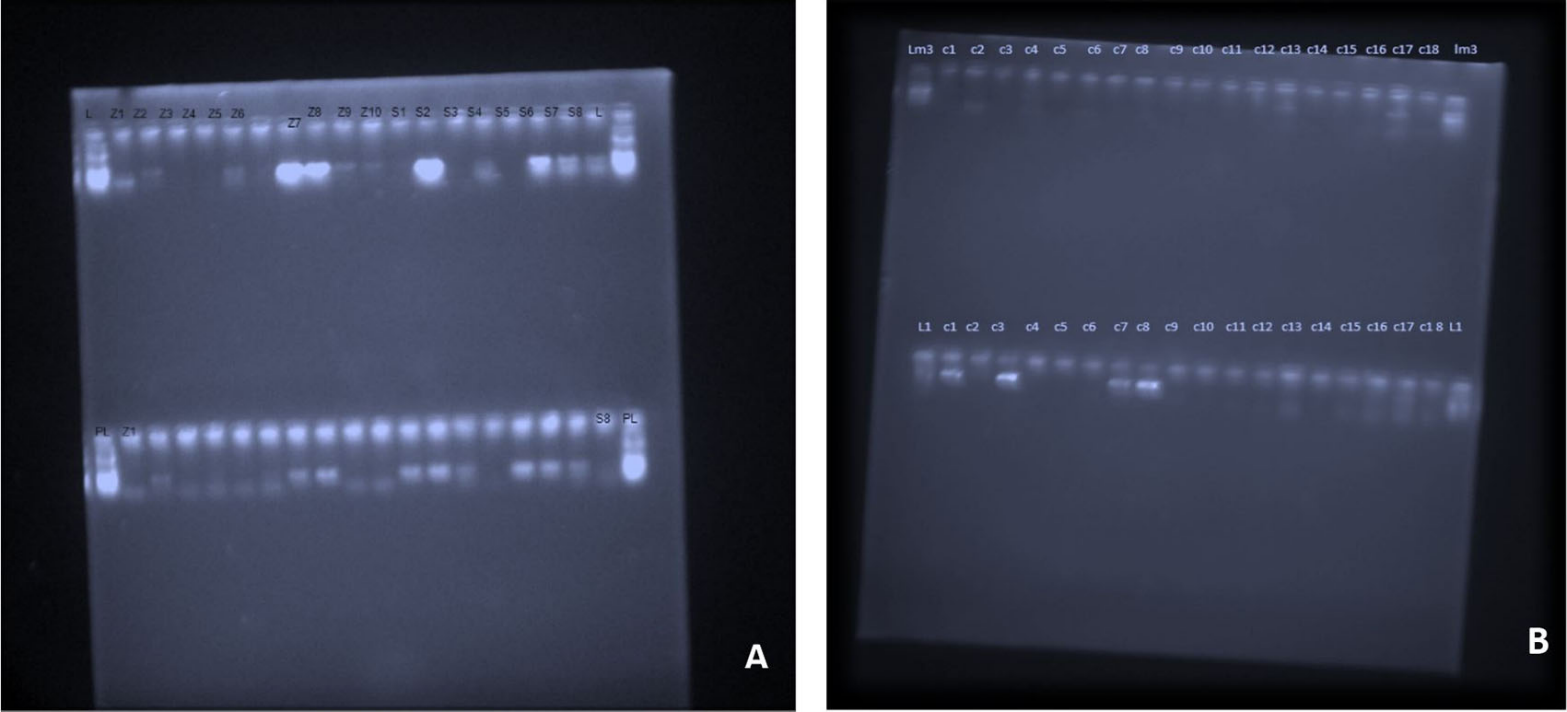
PCR Gel documentation; A, Pre-operative detection frequency of *A.a, T.f, F.n and P.g;* B,Post-operative identification of organisms at 3 months.

Regarding microbiological analysis, the detection frequencies of the 4 major putative periodontal pathogens were analysed. The detection frequencies of *P.g, A.a, T.f, F.n* were significantly lower at 3 months ( Figure 7). Even though the detection frequency lowered at the end of the study for all the pathogens, there was no statistical significance in any of the groups from baseline to 3 months. This may be due to the fact that the sample size for the present study was limited and for analysis of non-parametric variable such as the absence or presence of micro-organisms, a bigger sample size is necessary to demonstrate the effectiveness of the treatment modality.

No intergroup association was observed with respect to the presence of bacteria. Similar results were reported by Chondros et al. (2009), ^18^ Polansky et al. (2009), ^23^ De Micheli et al. (2011) ^24^ and Giannopoulou et al. (2012). ^25^ Polansky and group tested for P. gingivalis, T. forsythia and T. denticola at baseline and at 3 months. The levels of prevotella species (P.g) had significantly come down in both the groups. No significant reductions of T. forsythia and T. denticola were observed in either groups. Moreover, DeMicheli and group (2011) did not observe any additional reduction in the bacterial species in the test group (diode laser with SRP). Similar to the present study where the detection frequency of each organism tested, reduced significantly at 3 months, a systematic review of 17 randomised controlled clinical trials, by Akram et al. (2016) ^26^concluded that adjunctive PDT application led to a reduction of the following species *A.a, P.g, T. f, and T.d* equal to the control group (SRP alone). This was in line with another study by Cappuyns et al. (2011) ^19^ who demonstrated that detection frequency of *P.g, T.f* and *T.d* significantly reduced 2 and 6 months following intervention in all the groups that is SRP, DSL and PDT and that one procedure was not superior to the other.

The present study employed two sittings of PDT application similar to an earlier study by Segarra (2017) that have shown at least two applications of PDT is necessary to achieve the expected outcome. ^27^ On the contrary a study by Müller Campanile et al. (2015) demonstrated that the number of applications is not proportional to better outcomes of PDT. ^28^ Although there are not many systematic reviews to authenticate the statement, most of the available literature regarding the number of sittings of PDT emphasize of multiple sittings of atleast 2 or more than 2 to attain the desired effect ^29^^.^^30^ Maybe future trials can implement more than 2 sittings for treating residual pockets.

It is noteworthy to consider other limitations of the present study. Firstly, a bigger sample size is necessary to validate the use of indocyanine based PDT in maintenance patients. The time period taken as 3 months for the duration of the study provided a short term overview about the effect of different treatment modalities. A longer study period can be implied in future studies. There could be chances that the periodontopathogens could translocate from one niche to another within the oral cavity- which is known as intraoral translocation. This may explain the variable results obtained within the same patients from different treatment modalities.

## Conclusion

To date, successful application of antimicrobial photodynamic therapy remains conflicting and insufficient. This is because of the variation in methodology, poorly defined treatment parameters and the lack of a standard protocol in form of dosimetry or appropriate illumination devices. The present study does not show additional benefits of PDT over SRP; however, it does not negate the role of PDT with indocyanine green. Improvement was seen in the test and control group alike at the termination of the study period, though not statistically significant. Hence, future investigations should aim at standardising the treatment protocols of LASER, utilising indocyanine green in treatment of residual pockets, in a larger population. Furthermore, a quantitative analysis of the micro-organisms should be intended which would give us an enhanced understanding and prove the efficacy of ICG based antimicrobial photodynamic therapy.

## Data Availability

Figshare: Underlying data for “Indocyanine green based antimicrobial photodynamic therapy as an adjunct to non-surgical periodontal treatment in periodontal maintenance patients: a clinico-microbiological study”. https://doi.org/10.6084/m9.figshare.23573952. ^31^ The project contains the following underlying data:
1. Data file 1: Master chart containing raw data of both test group and control group at baseline and at 3 months (Table containing the raw data of the study) Figshare: Extended data for “Indocyanine green based antimicrobial photodynamic therapy as an adjunct to non-surgical periodontal treatment in periodontal maintenance patients: a clinico-microbiological study”. https://doi.org/10.6084/m9.figshare.23574150. ^32^ The project contains the following underlying data:
1. Case Performa 2. Informed consent form in English 3. Ethical Committee Approval letter Figshare: CONSORT check list and flow chart for “Indocyanine green based antimicrobial photodynamic therapy as an adjunct to non-surgical periodontal treatment in periodontal maintenance patients: a clinico-microbiological study”, https://doi.org/10.6084/m9.figshare.23574162. ^33^ Data are available under the terms of the Creative Commons Attribution 4.0 International license (CC-BY 4.0).
