# Supplementary material for: Indocyanine green based antimicrobial photodynamic therapy as an adjunct to non-surgical periodontal treatment in periodontal maintenance patients: a clinico-microbiological study
Source: F1000Res. 2024 Aug 20;12:949. Originally published 2023 Aug 7. [Version 2] doi: 10.12688/f1000research.133230.2 (PMC11409656; doi:10.12688/f1000research.133230.2)
Supplement: Peer review history

# Reviewer report: Reviewer response for version 2

**DOI:** 10.5256/f1000research.170408.r322362

**Published:** 2024-09-13

**Authors:** Biju Thomas

**Affiliations:** 1 A.B. Shetty Memorial Institute of Dental Science , Nitte University, Mangaluru, India

Indocyanine green based antimicrobial photodynamic therapy as an adjunct to non-surgical periodontal treatment in periodontal maintenance patients: a clinico-microbiological study. This split mouth study with 24 participants is well designed to evaluate the efficacy of photodynamic therapy as an adjunct in periodontal disease management. The study is novel in the use of  indocyanine green as the photosensitizer . The methodology and treatment protocol are clear. Results are clearly documented. The limitations of the study like possible intra oral translocation is highlighted. This is a novel study on a topic that has shown conflicting results.

Is the work clearly and accurately presented and does it cite the current literature?

Yes

If applicable, is the statistical analysis and its interpretation appropriate?

Yes

Are all the source data underlying the results available to ensure full reproducibility?

Yes

Is the study design appropriate and is the work technically sound?

Yes

Are the conclusions drawn adequately supported by the results?

Yes

Are sufficient details of methods and analysis provided to allow replication by others?

Yes

Reviewer Expertise:

stem cells applications in periodontal therapy, periodontal diseases management etc

I confirm that I have read this submission and believe that I have an appropriate level of expertise to confirm that it is of an acceptable scientific standard.

# Reviewer report: Reviewer response for version 2

**DOI:** 10.5256/f1000research.170408.r318749

**Published:** 2024-09-12

**Authors:** Anupama Rao

**Affiliations:** 1 Yenepoya Dental college, Mangalore, India

Authors have meticulously worked on study design methodology. Results are appropriately explained with proper literature support. Recent Systematic review finding has been included in the discussion part. Results are not manipulated. this articles gives future direction and also speaks about limitations of the present lack of standardization of diode laser protocol.

Is the work clearly and accurately presented and does it cite the current literature?

Yes

If applicable, is the statistical analysis and its interpretation appropriate?

Yes

Are all the source data underlying the results available to ensure full reproducibility?

Yes

Is the study design appropriate and is the work technically sound?

Yes

Are the conclusions drawn adequately supported by the results?

Yes

Are sufficient details of methods and analysis provided to allow replication by others?

Yes

Reviewer Expertise:

Microbiology, periodontal disease diagnosis and treatment

I confirm that I have read this submission and believe that I have an appropriate level of expertise to confirm that it is of an acceptable scientific standard.

# Reviewer report: Reviewer response for version 1

**DOI:** 10.5256/f1000research.146209.r222649

**Published:** 2024-07-01

**Authors:** Anurag Satpathy

**Affiliations:** 1 Department of Periodontics and Oral Implantology, Siksha 'O' Anusandhan University, Bhubaneswar, India

Authors have carried out the study "Indocyanine green based antimicrobial photodynamic therapy as an adjunct to non-surgical periodontal treatment in periodontal maintenance patients"

 While PDT is an accepted treatment modality and research continues to unravel the inconsistencies in the evidence, there are several areas in the present study where there is deficiency and lack of clarity.

 1. The timeline of treatment and its interval needs to be clarified. What was the time gap between the SRP and sample collection?

 2. Authors state that the inclusion criteria is greater than 4 mm probing depth. However, the PD in both groups at baseline is less than 3 mm.

 3. The McNemars test results are not presented

 4. Split mouth design with microbial assessment comparison between treatment and control has the chances of relocation of microbes

 5. PDT protocol is unclear.

Is the work clearly and accurately presented and does it cite the current literature?

Partly

If applicable, is the statistical analysis and its interpretation appropriate?

Partly

Are all the source data underlying the results available to ensure full reproducibility?

Partly

Is the study design appropriate and is the work technically sound?

No

Are the conclusions drawn adequately supported by the results?

No

Are sufficient details of methods and analysis provided to allow replication by others?

No

Reviewer Expertise:

Periodontal Regeneration, Periodontal Microbiology, Autologus platelet concentrate, Periodontal Medicine

I confirm that I have read this submission and believe that I have an appropriate level of expertise to state that I do not consider it to be of an acceptable scientific standard, for reasons outlined above.

# Response

**Published:** 2024-08-14

**Authors:** DEEPA KAMATH

**Affiliations:** Manipal University, India

1. The timeline of treatment and its interval needs to be clarified. What was the time gap between the SRP and sample collection?

***Response : **Changes done. PDT application was done at baseline and again after 14 days. However, the clinical and microbiological assessment was done at baseline and after 3 months only*

 2. Authors state that the inclusion criteria is greater than 4 mm probing depth. However, the PD in both groups at baseline is less than 3 mm.

***Response: **The results were calculated as follows-*

*Sum of pocket depth of test/control sites of an individual divided by total number of sites of the selected teeth.*

*However, the calculations are re-checked and changes in the values are made.*

 3.The McNemars test results are not presented

***Response: **Changes done. Table 4 has been added showing the results of Mc Nemar P Test. The same had been depicted graphically in the old version.*

 4. Split mouth design with microbial assessment comparison between treatment and control has the chances of relocation of microbes

***Response : **This has been mentioned as one of the drawbacks of the study. Besides, there are no statistical tests to detect carry-across effect. However, split mouth designs do provide with better standardisation in terms of reduced variability.*

*5. *PDT protocol is unclear.

***Response: **Changes done. a-PDT was carried out at baseline after completion of full mouth SRP of the selected individuals and repeated after 14 days. The preparation of dye was according to the instructions provided in the product manual.*

# Reviewer report: Reviewer response for version 1

**DOI:** 10.5256/f1000research.146209.r266858

**Published:** 2024-07-01

**Authors:** Swati Setty

**Affiliations:** 1 SDM College of Dental Sciences and Hospital, Dharwad, Karnataka, India

This manuscript has adopted a proper study design with the support of current literature. The need for research on photodynamic therapy has been described. The methodology of the study is adequate. The interpretation of the results is appropriate. Discussion includes the related literature of the study. Limitations have been explained correlating with the current evidence. The requirement for further research has been stated clearly.

Is the work clearly and accurately presented and does it cite the current literature?

Yes

If applicable, is the statistical analysis and its interpretation appropriate?

Yes

Are all the source data underlying the results available to ensure full reproducibility?

Yes

Is the study design appropriate and is the work technically sound?

Yes

Are the conclusions drawn adequately supported by the results?

Yes

Are sufficient details of methods and analysis provided to allow replication by others?

Yes

Reviewer Expertise:

NA

I confirm that I have read this submission and believe that I have an appropriate level of expertise to confirm that it is of an acceptable scientific standard.
